# National Mortality Patterns of Cancer Cachexia in Gastrointestinal Malignancies in the United States: A Centers for Disease Control and Prevention Wide-Ranging Online Data for Epidemiologic Research (CDC WONDER) Analysis (2018–2023)

**DOI:** 10.7759/cureus.106288

**Published:** 2026-04-01

**Authors:** Mohammad Alali, Mohammad Alkhaleel Alomar, Mouhammad Alyounes

**Affiliations:** 1 Internal Medicine, Henry Ford Hospital, Macomb, USA; 2 General Medicine, Alexandria Faculty of Medicine, Alexandria, EGY

**Keywords:** cancer cachexia, cdc wonder, demographic disparities, epidemiology, gastrointestinal malignancies, mortality trends, palliative oncology, population-based study

## Abstract

Background: Cancer cachexia is a multifactorial metabolic syndrome characterized by progressive weight loss, skeletal muscle wasting, anorexia, and systemic inflammation that cannot be fully reversed by conventional nutritional support. It frequently complicates advanced malignancy and is particularly prevalent in gastrointestinal cancers. Despite its clinical significance, population-level data describing mortality patterns involving cachexia among patients with gastrointestinal malignancies remain limited. Objective: This study aims to evaluate national mortality trends and demographic disparities in individuals where cachexia was identified as a contributing cause of death among those with gastrointestinal malignancies in the United States. Materials and methods: We conducted a retrospective population-based cross-sectional study using the Centers for Disease Control and Prevention Wide-Ranging Online Data for Epidemiologic Research (CDC WONDER) Multiple Cause of Death database from 2018 to 2023. Deaths were included if gastrointestinal malignancy (ICD-10 codes C15-C26) was listed as the underlying cause of death and cachexia (ICD-10 code R64) was listed as a contributing cause. Annual mortality counts and crude mortality rates per 100,000 population were calculated and stratified by sex, race, and U.S. census region.

Results: A total of 3,888 deaths were identified during the study period, corresponding to an overall crude mortality rate of 0.20 per 100,000 population. Annual mortality remained relatively stable, ranging from 619 deaths in 2021 to 699 deaths in 2018, with crude mortality rates between 0.19 and 0.21 per 100,000. Men accounted for the majority of deaths (61.7%) and demonstrated higher mortality rates compared with women (0.24 vs 0.15 per 100,000). By race, White individuals demonstrated the highest crude mortality rate (0.22 per 100,000), followed by Black (0.18) and Asian (0.13) populations. Regional variation was also observed, with the highest mortality rates occurring in the Western United States (0.25 per 100,000), followed by the South (0.20), Midwest (0.18), and Northeast (0.13).

Conclusions: Cachexia is documented in a measurable but likely underestimated proportion of deaths among patients with gastrointestinal malignancies in the United States. Significant sex, racial, and geographic disparities were observed in mortality patterns. These findings highlight the potential underrecognition of cancer cachexia and emphasize the importance of improved clinical awareness, documentation, and early integration of multidisciplinary supportive care strategies for patients with advanced gastrointestinal cancers.

## Introduction

Cancer cachexia is a multifactorial metabolic syndrome characterized by involuntary weight loss, skeletal muscle wasting, anorexia, and systemic inflammation that cannot be fully reversed by conventional nutritional support [1]. Cachexia represents one of the most clinically significant complications of advanced malignancy. It has been estimated to affect up to 80% of patients with late-stage cancer while contributing directly to nearly 20% of cancer-related deaths [2].

Gastrointestinal (GI) malignancies, including pancreatic, gastric, hepatic, and colorectal cancers, are particularly associated with the development of cancer cachexia [3]. The high prevalence of cachexia in these malignancies is thought to result from complex tumor-host metabolic interactions, chronic systemic inflammation, and alterations in energy balance driven by inflammatory cytokines and tumor-derived factors [4]. These processes lead to progressive skeletal muscle loss, functional decline, and reduced tolerance to oncologic therapies [5].

Despite its substantial clinical impact, cachexia remains underrecognized and inconsistently documented in clinical practice [6]. Prior epidemiologic investigations examining cachexia in cancer populations have largely been derived from single-center cohorts or disease-specific studies, limiting their generalizability to broader populations [7]. Population-level analyses evaluating national mortality patterns involving cachexia among patients with GI malignancies remain limited.

Understanding national trends and demographic disparities in cachexia-associated mortality may provide insights into potential differences in disease burden, healthcare access, and recognition of cachexia in clinical practice. Additionally, identifying patterns across demographic groups may help inform strategies for earlier recognition and integration of supportive and palliative care services for patients with advanced malignancies complicated by cachexia [8].

Given the particularly high prevalence of cachexia among GI cancers, this population represents an important group for evaluating national mortality patterns related to this syndrome. We therefore performed a population-based analysis using the Centers for Disease Control and Prevention Wide-Ranging Online Data for Epidemiologic Research (CDC WONDER) Multiple Cause of Death database to evaluate mortality involving cachexia among individuals dying from GI malignancies in the United States between 2018 and 2023. We hypothesized that mortality involving cachexia would demonstrate measurable demographic and geographic disparities across the United States.

## Materials and methods

Study design and data source

We conducted a retrospective, population-based, cross-sectional study using mortality data from the CDC WONDER Multiple Cause of Death database [9]. This publicly accessible dataset compiles information from U.S. death certificates filed in all 50 states and the District of Columbia, including demographic characteristics and underlying and contributing causes of death coded using the International Classification of Diseases, Tenth Revision (ICD-10) [10]. The CDC WONDER database is widely used in epidemiologic research to evaluate national mortality trends and population-level health disparities.

Data processing and analysis

Data extraction was performed using the CDC WONDER online interface. Additional analyses and figure generation were conducted using Microsoft Excel (Microsoft Corp., Redmond, WA, USA). No additional exclusion criteria were applied beyond database-defined constraints.

Study population

Deaths occurring between January 1, 2018, and December 31, 2023, were included in this analysis. Eligible records were identified using the ICD-10 coding system [10], specifying GI malignancies as the underlying cause of death and cachexia as a contributing cause of death. GI malignancies were defined using ICD-10 codes C15-C26, which include malignant neoplasms of the esophagus, stomach, small intestine, colon, rectum, liver, gallbladder, pancreas, and other digestive organs. Cachexia was identified using the ICD-10 code R64, listed among the contributing causes of death. Records meeting both criteria were included in the final analytic dataset, enabling identification of deaths in which GI malignancy was the primary cause. At the same time, cachexia was documented as a contributing condition. No additional exclusion criteria were applied beyond the dataset's predefined inclusion parameters.

Variables and stratification

Mortality data were stratified according to several demographic and geographic variables available within the CDC WONDER database, including year of death (2018-2023), sex (male or female), race categorized using the CDC “Single Race” classification (White, Black or African American, Asian, American Indian/Alaska Native, and Other), and U.S. census region categorized as Northeast, Midwest, South, and West. These variables were selected to evaluate potential demographic and geographic disparities in mortality patterns involving cancer cachexia among individuals with GI malignancies.

Mortality measures

Annual death counts and crude mortality rates per 100,000 population were calculated using population denominators provided within the CDC WONDER database. Crude mortality rates were calculated to allow comparison of mortality patterns across demographic groups and geographic regions. Temporal trends in mortality were evaluated descriptively across the study period. Because annual mortality variation was relatively small and the study was designed as a descriptive epidemiologic analysis, formal inferential statistical testing was not performed.

Data suppression and confidentiality

In accordance with CDC data-use policies, death counts of fewer than 10 are suppressed within the CDC WONDER database to protect confidentiality. Suppressed values were handled in accordance with CDC reporting guidelines and were excluded from subgroup analyses when necessary.

Ethical considerations

All authors have confirmed that this study involved human participants using publicly available, de-identified mortality data from the CDC WONDER database. Because the dataset contains no identifiable information, institutional review board approval was not required, and informed consent was waived.

## Results

Overall mortality

Between 2018 and 2023, a total of 3,888 deaths were identified in the United States in which GI malignancy was listed as the underlying cause of death, and cachexia was documented as a contributing condition. The overall crude mortality rate during the study period was 0.20 per 100,000 population. These findings indicate that although cachexia is frequently associated with advanced malignancy, it was documented in a relatively small proportion of deaths among individuals with GI cancers.

Temporal trends

**Figure 1:**
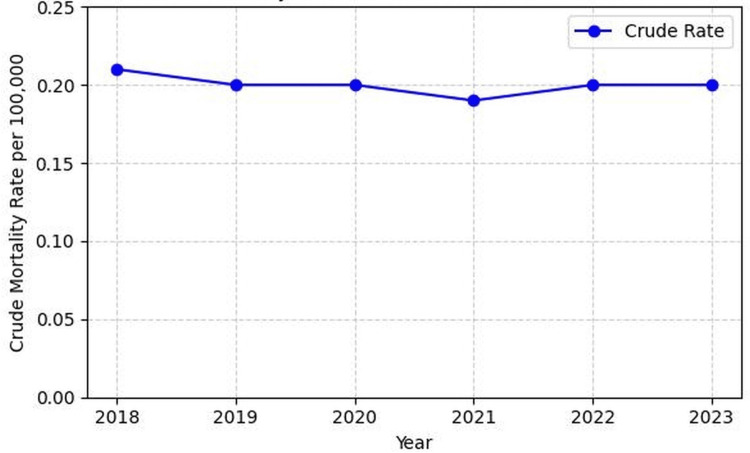
Annual crude mortality rate involving cachexia among individuals with GI malignancies in the United States (2018–2023) The figure illustrates the annual crude mortality rate per 100,000 population for deaths in which GI malignancy (ICD-10 codes C15–C26) was listed as the underlying cause of death and cachexia (ICD-10 code R64) was documented as a contributing condition. Mortality rates remained relatively stable during the study period, ranging from 0.19 to 0.21 per 100,000 population. ICD-10: International Classification of Diseases, Tenth Revision, GI: gastrointestinal

Annual mortality involving cachexia among individuals with GI malignancies remained relatively stable during the study period. The number of deaths ranged from 619 in 2021 to 699 in 2018. Corresponding crude mortality rates varied only slightly across years, ranging from 0.19 to 0.21 per 100,000 population. Overall mortality declined modestly by approximately 7% over the study period, though no consistent directional trend was observed (Figure 1).

Sex differences

**Figure 2:**
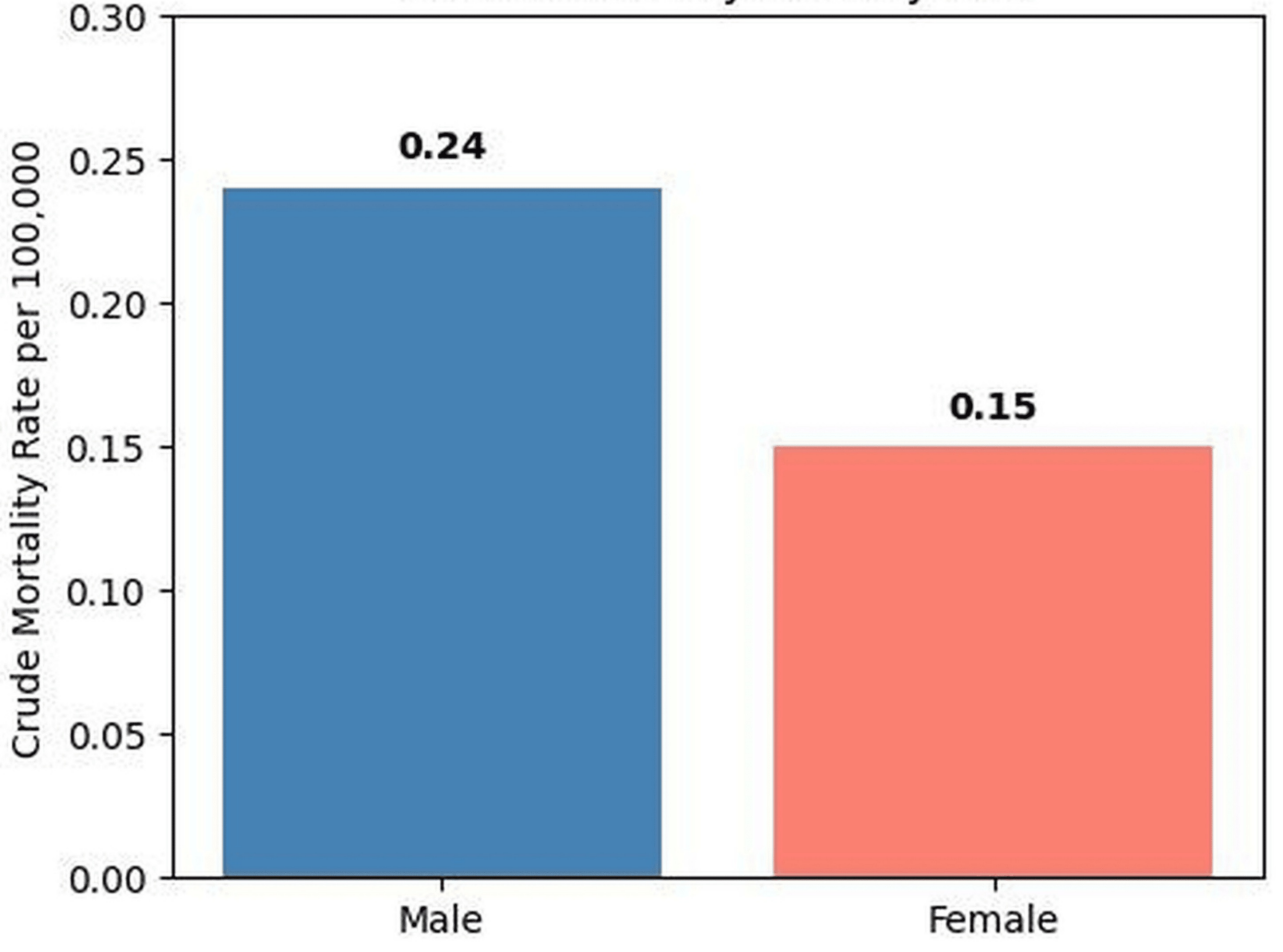
Crude mortality rate involving cachexia among individuals with GI malignancies by sex in the United States (2018–2023) The figure shows crude mortality rates per 100,000 population among individuals with GI malignancies where cachexia (ICD-10 code R64) was listed as a contributing cause of death. Mortality was higher among males (0.24 per 100,000) compared with females (0.15 per 100,000), representing approximately 65% higher mortality in men. ICD-10: International Classification of Diseases, Tenth Revision, GI: gastrointestinal

Notable differences in mortality were observed between males and females. Men accounted for 2,400 deaths (61.7%) with a crude mortality rate of 0.24 per 100,000 population, while women accounted for 1,488 deaths (38.3%) with a crude mortality rate of 0.15 per 100,000 population. Overall, mortality involving cachexia among individuals with GI malignancies was approximately 65% higher in males compared with females (Figure 2).

Racial differences

**Figure 3:**
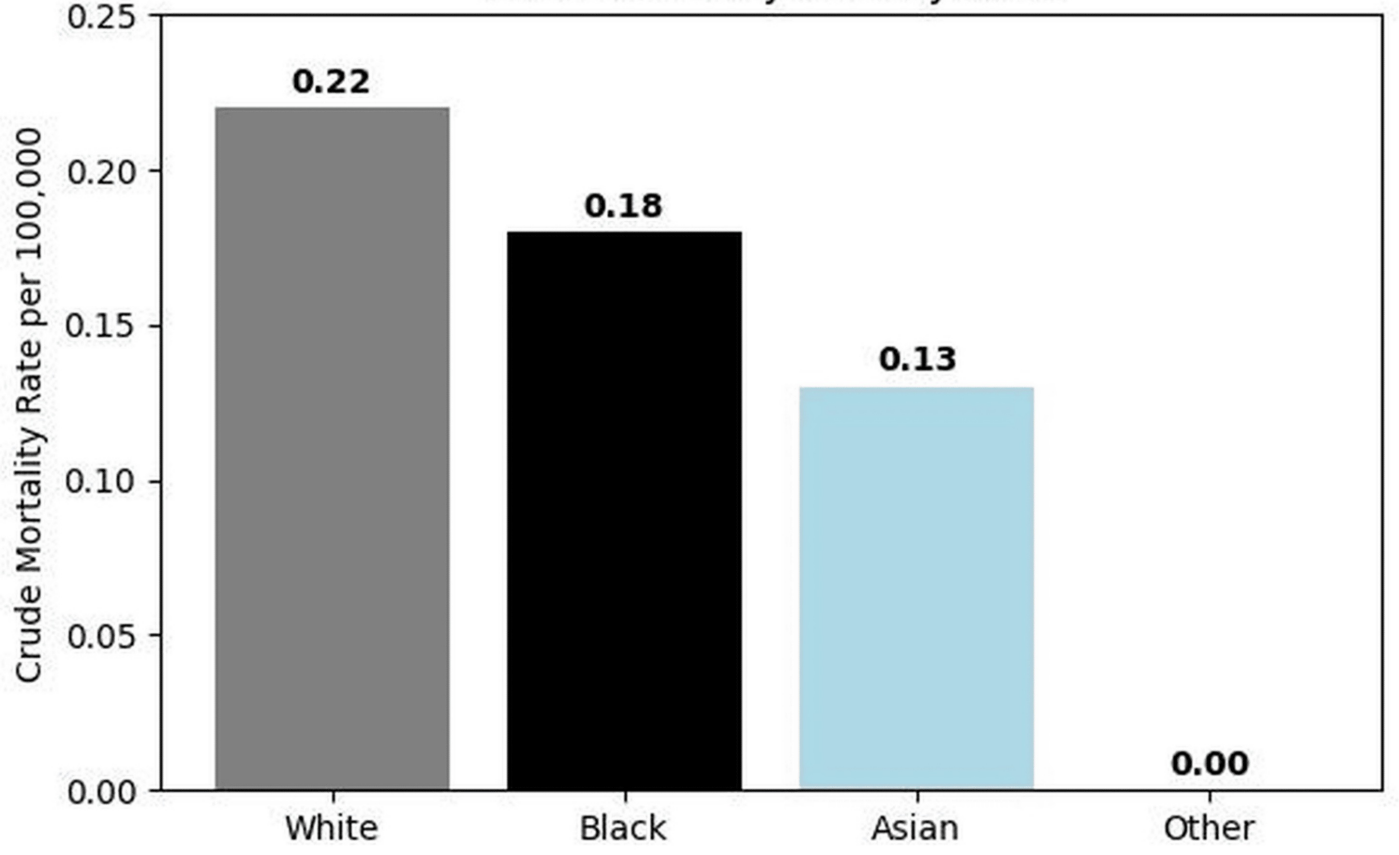
Crude mortality rate involving cachexia among individuals with GI malignancies by race in the United States (2018–2023) The figure illustrates crude mortality rates per 100,000 population among individuals with GI malignancies where cachexia (ICD-10 code R64) was documented as a contributing cause of death. White individuals demonstrated the highest crude mortality rate (0.22 per 100,000), followed by Black individuals (0.18 per 100,000) and Asian individuals (0.13 per 100,000). Mortality rates among other racial categories were minimal in the dataset. ICD-10: International Classification of Diseases, Tenth Revision, GI: gastrointestinal

Mortality patterns differed across racial groups. White individuals accounted for the majority of deaths (3,248 deaths; 83.5%) and demonstrated the highest crude mortality rate of 0.22 per 100,000 population. Black individuals had a crude mortality rate of 0.18 per 100,000, while Asian individuals demonstrated a lower rate of 0.13 per 100,000 (Figure 3). These findings suggest measurable racial variation in mortality involving cachexia among individuals with GI malignancies.

Regional variation

**Figure 4:**
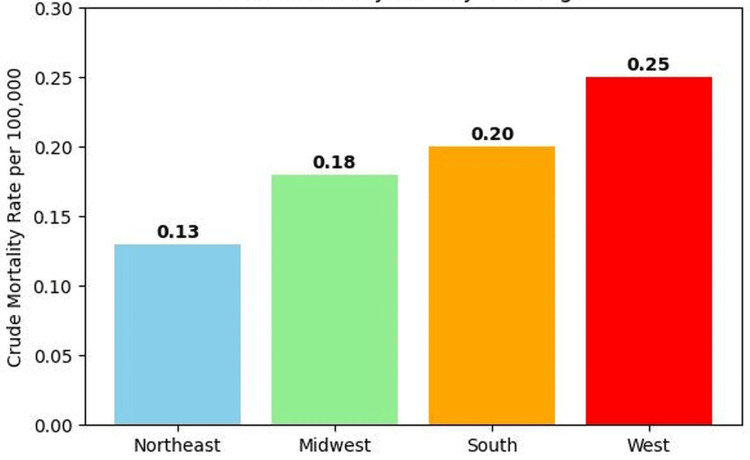
Crude mortality rate involving cachexia among individuals with GI malignancies by U.S. Census Region (2018–2023) The figure displays crude mortality rates per 100,000 population stratified by U.S. census region. Mortality rates were highest in the Western United States (0.25 per 100,000), followed by the South (0.20 per 100,000), Midwest (0.18 per 100,000), and Northeast (0.13 per 100,000), suggesting potential geographic variation in disease burden, healthcare access, or clinical documentation practices. ICD-10: International Classification of Diseases, Tenth Revision, GI: gastrointestinal

Geographic differences in mortality patterns were observed across U.S. census regions. The Western United States had the highest crude mortality rate (0.25 per 100,000 population), followed by the South (0.20 per 100,000), the Midwest (0.18 per 100,000), and the Northeast (0.13 per 100,000) (Figure 4). These regional variations suggest potential differences in disease burden, healthcare access, or clinical documentation practices across geographic regions.

## Discussion

In this national population-based analysis of U.S. mortality data, we identified 3,888 deaths between 2018 and 2023 in which GI malignancy was listed as the underlying cause of death and cachexia was documented as a contributing condition. Although the absolute mortality rate remained relatively low at approximately 0.20 per 100,000 population, our findings demonstrate measurable demographic and geographic differences in cachexia-associated mortality among patients with GI cancers.

Cancer cachexia is a complex metabolic syndrome characterized by systemic inflammation and progressive muscle wasting [4]. In GI malignancies, these processes contribute to functional decline, reduced treatment tolerance, and increased mortality risk [3,5].

The higher mortality observed among men in this study is consistent with prior observations of sex-based differences in cancer incidence, body composition, and inflammatory responses [11]. Male patients generally have greater baseline skeletal muscle mass, which may lead to more pronounced detectable muscle loss during cancer progression, potentially increasing the clinical recognition or documentation of cachexia. Additionally, differences in healthcare utilization and cancer screening patterns between men and women may influence the stage at diagnosis and subsequent disease trajectory.

Racial differences in cachexia-associated mortality were also observed, with White individuals demonstrating the highest crude mortality rates in our analysis. These patterns may reflect a combination of factors, including differences in cancer incidence, socioeconomic determinants of health, access to oncology and supportive care services, and potential variations in diagnostic coding practices [12]. Previous studies have demonstrated that disparities in cancer outcomes are often influenced by structural determinants of health, such as healthcare access, geographic location, and socioeconomic status, rather than intrinsic biologic differences alone [13].

Regional variation in mortality patterns was also evident, with the highest crude mortality rates observed in the Western United States. Geographic differences in healthcare infrastructure, access to specialized cancer centers, availability of palliative care services, and regional coding practices may contribute to these findings [12,13]. Differences in population demographics and regional cancer incidence patterns may also influence observed mortality distributions across census regions.

An important finding of this study is the relatively low overall mortality rate involving documented cachexia despite the well-established high prevalence of cachexia among patients with advanced malignancies [2]. This discrepancy likely reflects substantial underrecognition and underdocumentation of cachexia in clinical practice and on death certificates. Cachexia is often considered an expected complication of advanced cancer and may therefore be omitted as a contributing cause of death despite its significant impact on patient outcomes [14]. Prior research has demonstrated that weight loss and nutritional decline in cancer patients are frequently underreported in administrative datasets and death records [8]. Therefore, the observed mortality rates likely underestimate the true burden of cancer cachexia among patients with GI malignancies.

From a clinical perspective, improved recognition of cancer cachexia has important implications for patient management. Early identification of cachexia may prompt multidisciplinary supportive interventions, including nutritional counseling, appetite stimulation therapies, physical rehabilitation, and symptom-directed treatment strategies [15]. Additionally, recognition of cachexia may facilitate earlier referral to palliative care services, which have been shown to improve symptom control and quality of life for patients with advanced malignancies. Such interventions may not reverse the underlying metabolic syndrome but can significantly improve patient-centered outcomes and functional status.

Population-level analyses such as this study can provide valuable insight into the broader epidemiologic burden of cachexia across cancer populations. Identifying demographic and geographic patterns in mortality may help guide future research aimed at improving recognition, documentation, and management of cancer cachexia. Future studies incorporating longitudinal clinical data, cancer stage information, age-adjusted mortality rates, and proportionate mortality analyses using GI cancer deaths as a denominator may provide more clinically meaningful estimates of cachexia burden and better characterize its clinical trajectory.

This study has several limitations. Mortality data derived from death certificates are subject to potential misclassification and underreporting. Cachexia is frequently underdiagnosed and inconsistently documented, which likely leads to underestimation of its true prevalence. Additionally, the CDC WONDER database does not provide granular clinical variables such as cancer stage, treatment history, or nutritional assessments that may influence outcomes. Furthermore, the use of crude mortality rates without age adjustment may limit comparisons across demographic groups, as observed differences may partially reflect variations in population age structure rather than true differences in disease burden. Finally, the observational nature of this analysis precludes causal inference regarding the relationship between cachexia and mortality.

Despite these limitations, this study provides a national overview of mortality involving cachexia among patients with GI malignancies. Our findings highlight measurable demographic and geographic differences in cachexia-associated mortality and underscore the likely underrecognition of this syndrome in routine clinical practice. Improved awareness, earlier identification, and standardized documentation of cachexia may enhance supportive care strategies and inform future research aimed at improving outcomes for patients with advanced GI cancers.

## Conclusions

Cancer cachexia is documented in a measurable but likely underestimated proportion of deaths among individuals with gastrointestinal malignancies in the United States. In this national population-based analysis, mortality involving cachexia remained relatively stable between 2018 and 2023 but demonstrated notable differences across sex, racial groups, and geographic regions. These findings suggest that cancer cachexia may be underrecognized and inconsistently documented despite its well-established clinical impact in advanced malignancy. Improved awareness, earlier identification, and standardized documentation of cachexia may help facilitate timely supportive interventions and multidisciplinary care for patients with gastrointestinal cancers. Further research incorporating detailed clinical data is needed to better characterize the true burden of cachexia and to inform strategies aimed at improving outcomes and quality of life for affected patients.
